# CSN-CRL Complexes: New Regulators of Adipogenesis

**DOI:** 10.3390/biom15030372

**Published:** 2025-03-05

**Authors:** Dawadschargal Dubiel, Michael Naumann, Wolfgang Dubiel

**Affiliations:** Institute of Experimental Internal Medicine, Medical Faculty, Otto von Guericke University, Leipziger Str. 44, 39120 Magdeburg, Germany; naumann@med.ovgu.de

**Keywords:** COP9 signalosome (CSN), cullin-RING-ubiquitin ligases (CRLs), adipogenesis, cell cycle inhibitor p27^KIP^, obesity, treatment

## Abstract

Recent discoveries revealed mechanistic insights into the control of adipogenesis by the Constitutive Photomorphogenesis 9 Signalosome (CSN) and its variants, CSN^CSN7A^ and CSN^CSN7B^, which differ in the paralog subunits, CSN7A and CSN7B. CSN^CSN7A^ and CSN^CSN7B^ variants form permanent complexes with cullin-RING-ubiquitin ligases 3 and 4A (CRL3 and CRL4A), respectively. These complexes can be found in most eukaryotic cells and represent a critical reservoir for cellular functions. In an early stage of adipogenesis, mitotic clonal expansion (MCE), CSN-CRL1, and CSN^CSN7B^-CRL4A are blocked to ubiquitinate the cell cycle inhibitor p27^KIP^, leading to cell cycle arrest. In addition, in MCE CSN-CRL complexes rearrange the cytoskeleton for adipogenic differentiation and CRL3^KEAP1^ ubiquitylates the inhibitor of adipogenesis C/EBP homologous protein (CHOP) for degradation by the 26S proteasome, an adipogenesis-specific proteolysis. During terminal adipocyte differentiation, the CSN^CSN7A^-CRL3 complex is recruited to a lipid droplet (LD) membrane by RAB18. Currently, the configuration of the substrate receptors of CSN^CSN7A^-CRL3 on LDs is unclear. CSN^CSN7A^-CRL3 is activated by neddylation on the LD membrane, an essential adipogenic step. Damage to CSN/CUL3/CUL4A genes is associated with diverse diseases, including obesity. Due to the tremendous impact of CSN-CRLs on adipogenesis, we need strategies for adequate treatment in the event of malfunctions.

## 1. Introduction

The essential role of the Constitutive Photomorphogenesis 9 Signalosome (CSN) in adipogenesis was demonstrated more than a decade ago [1]. Recently, the significance of CSN’s interaction with cullin-RING-ubiquitin ligases (CRLs) to form permanent CSN-CRL complexes was recognized [1,2,3]. The complexes are used for diverse cellular functions, including adipogenesis [1]. Therefore, the main issue in this review is to illustrate the role of CSN^CSN7A^-CRL3 and CSN^CSN7B^-CRL4 particles in adipogenesis and their malfunction in the development of obesity. The significance of CSN-CRLs was discovered by Bennett et al. [2] and recently described in detail [4].

Most studies on adipogenic differentiation are undertaken with mouse 3T3-L1 preadipocytes [5]. In addition, human LiSa-2 cells [1] or mouse embryonic fibroblasts [5] can also be used as adipocyte differentiation models. Results on CSN-CRL complexes mostly come from LiSa-2 cells [1]. In mouse embryonic fibroblasts, some data on CSN-CRL complexes were confirmed [6]. In 3T3-L1 cells, recent data support findings on LiSa-2 cells in CSN-CRL complexes [7], and other data are scattered and will be compiled during this review.

**Figure 1:**
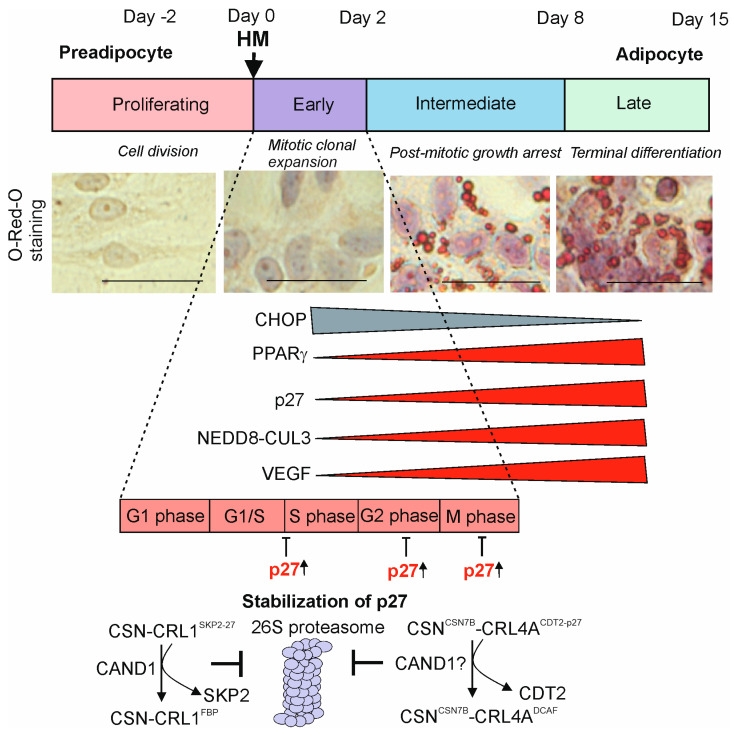
Stages of adipogenesis in LiSa-2 cells. Upon addition of a hormone mixture (HM) consisting of insulin, triiodothyronine, and cortisol, LiSa-2 preadipocytes differentiate over about fourteen days to mature adipocytes. Light microscopic images stained with O-Red-O (ORO) before HM (proliferating) and after HM, during mitotic clonal expansion (MCE); post-mitotic growth arrest and terminal adipocyte differentiation (TAD) are shown. Staining experiments are newly generated for this review. Bars represent 50 µm. Expression of proteins that decrease (CHOP) or increase (PPARγ, p27, neddylated CUL3, VEGF) is indicated. Cell cycle phases are demonstrated during MCE. The mechanism of p27 accumulation takes place by the cullin-associated and neddylation-dissociated 1 (CAND1)-dependent release of the appropriate substrate receptors (SRs) SKP2 from CSN^CSN1^-CRL1^SKP2-p27^ and CDT2 from CSN^CSN7B^-CRL4A^CDT2-p27^. Consequently, protein p27 is not ubiquitylated and not degraded by the 26S proteasome and accumulates [1].

Adipogenic differentiation is governed by a plethora of regulatory proteins; many are degraded by the ubiquitin–proteasome system (UPS) [8,9,10,11]. Differentiation of human liposarcoma LiSa-2 preadipocytes is stimulated by a hormone mixture (HM) consisting of insulin, triiodothyronine, and cortisol (Figure 1) [12].

Insulin is classically viewed as a promoter of adipogenesis as it increases the expression of cAMP-response element-binding protein (CREB) and peroxisome proliferator-activated receptor γ (PPARγ) [13]. Cortisol enhances adipogenic differentiation by upregulating CCAAT/enhancer-binding proteins (C/EBPs) [14] and triiodothyronine increases lipid droplet formation [15]. It is one of the most suitable models, possessing a high potential for in vitro adipogenesis. The process can be visualized by staining lipids with Oil Red O (ORO), which accumulates in LDs (Figure 1). In this review, we will discuss the role of CSN-CRL complexes in adipogenesis, which occurs in two major steps: mitotic clonal expansion (MCE) of preadipocytes and terminal adipocyte differentiation (TAD).

Recently, malfunctions of CSN-CRL complexes have been associated with obesity, the most serious public health threat because of its connection with metabolic syndromes like type 2 diabetes, dyslipidemia, hypertension, and cardiovascular diseases as well as cancer [16,17]. Obesity is an expansion of the white adipose tissue (WAT) by hyperplasia, when new adipocytes are formed from precursors during adipogenesis, and/or hypertrophia, the increase in size of existing adipocytes [8]. Normally, the main function of WAT is to store energy in the form of triglycerides. In addition, fat tissue acts as an endocrine organ by secreting a large number of hormones and cytokines called adipokines [18]. For example, leptin, adiponectin, vascular endothelial growth factor (VEGF), and apelin are adipokines secreted by adipocytes and involved in energy homeostasis, inflammation, and insulin resistance [19]. Overexpression of adipokines because of obesity increases the risk of metabolic diseases and cancer. Therefore, for prevention and treatment of obesity, it is important to study the underlying CSN-CRL mechanisms of adipose tissue formation.

## 2. Composition of CSN-CRL Complexes

**Figure 2:**
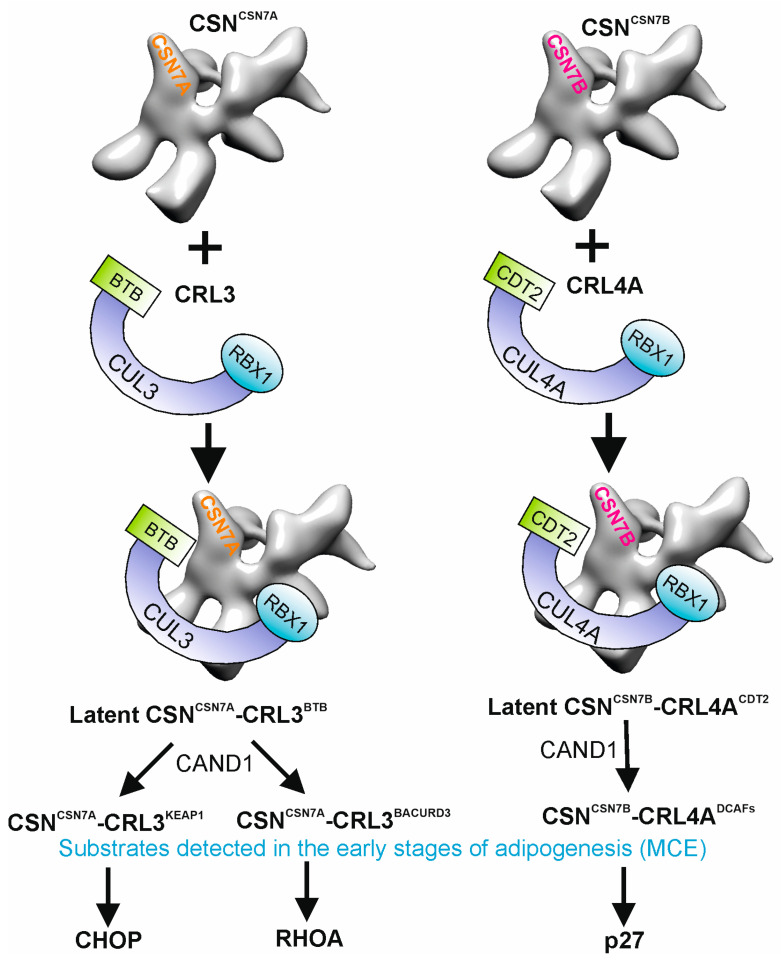
Preferential binding of CSN^CSN7A^ to CRL3^BTB^ and of CSN^CSN7B^ to CRL4A^DCAFs^ to form permanent complexes and substrates detected in mitotic clonal expansion (MCE). During early phases of adipogenesis, CAND1 exchanges CSN^CSN7A^-CRL^BTB^ substrate receptors to KEAP1 and to BACURD3 as well as CSN^CSN7B^-CRL4A^CDT2^ to other DDB1- and CUL4-associated factors (DCAFs). In MCE, CSN^CSN7A^-CRL3^KEAP1^ ubiquitylates CHOP, CSN^CSN7A^-CRL3^BACURD3^ is responsible for RHOA ubiquitylation, and CSN^CSN7B^-CRL4^CDT2^ ubiquitylates p27 for degradation by the 26S proteasome.

The core CSN in *humans* consists of eight subunits (CSN1-CSN8). It occurs as variants which differ by paralog subunits [20,21]. Two variants in human cells are CSN^CSN7A^ and CSN^CSN7B^, characterized by the paralog subunits CSN7A and CSN7B. In addition, there are CSN^CSN8A^ and CSN^CSN8B^ variants in *humans* and CSN^CSN5A^, CSN^CSN5B^, CSN^CSN6A^, and CSN^CSN6B^ in plants [20], which will not be addressed in this review. While both CSN7A and CSN7B subunits have identical Proteasome lid-CSN-Initiation factor 3 (PCI) domains, they differ in their N- and C-termini. Overall, they possess an identity of about 60% [1]. CSN^CSN7A^ and CSN^CSN7B^ variants are characterized by specific interaction partners. CSN^CSN7A^ preferentially binds CRL3 and CSN^CSN7B^ to CRL4A/B. Stable CSN^CSN7A^-CRL3^BTB^ (BTB-Bric-a-brac, tramtrack and broad complex/Pox virus and Zinc finger proteins) and CSN^CSN7B^-CRL4A/B^DCAF^ (DCAF-DDB1 and CUL4-associated factors) can be found in mammalian cells in a latent form and are therefore called latent CSN-CRL complexes (Figure 2) [1].

Although neddylation–deneddylation of CRL complexes has impact on ubiquitylation by CRLs [22], it does not influence the stability of latent CSN-CRL complexes as demonstrated by the inhibitors MLN4924, the neddylation inhibitor [23], and CSN5i-3, a specific inhibitor of CSN-mediated deneddylation [22], respectively [1]. The existence of CSN-CRL complexes in different cells independent of MLN4924 has been described and a model of CRL dynamics was suggested, where the abundance of components drive CRL network organization [2]. In the current model, CRL dynamics is regulated by cycles of deneddylation and cullin-associated and neddylation-dissociated 1 (CAND1) to adapt CRLs to fluctuations in substrate availability [24,25]. Based on new data, we suggest that both models exist. Latent CSN-CRLs are already present under steady-state conditions and as a main reservoir available in most cells for diverse cell functions [1]. However, under specific conditions like adipogenesis, a fast exchange of substrate receptors (SRs) requires CAND1 cycles [26].

CSN-CRL complexes possess a slow turnover and interact with a multitude of additional proteins. First of all, deubiquitylase enzymes (DUBs) are associated with the CSN deneddylase forming multi-DUB complexes [27]. While, in human cells, USP15 specifically binds to CSN7A [28] and consequently to the CSN^CSN7A-USP15^-CRL3^BTB^ complex [1], USP48 is associated with CSN1 [29] but interacts mostly with CSN^CSN7B-USP48^-CRL4A/B [1]. The CSN-associated DUBs contribute to the stabilization of the target proteins [30], although the exact mechanism has not yet been discovered.

Another CSN-CRL-interacting protein is the cell cycle inhibitor p27^KIP1^ (p27), most of which is associated with CSN^CSN7B^-CRL4A [1,31]. Since there is no specific binding site for p27 in the complex, it has been concluded that p27 binds to its SR, CDC10-dependent transcript 2 (CDT2), in the complex CSN^CSN7B^-CRL4A^CDT2-p27^. The additional two highly important candidates of the CSN-CRL interactome are the small GTPase RAB18 and caveolin 1 (CAV1). Under non-stimulatory cell conditions, immunoprecipitations reveal predominant binding of RAB18 and CAV1 to CSN^CSN7A^-CRL3^BTB^. However, there is also binding to CSN^CSN7B^-CRL4A/B. Under conditions of adipogenesis in LiSa-2 cells, RAB18 is phosphorylated, which promotes exclusive binding to CSN^CSN7A^-CRL3^BTB^ [1].

## 3. Functions of CSN-CRL Complexes in Mitotic Clonal Expansion

White adipose tissue (WAT) is the main adipose tissue in humans, representing 10–15% and 25% of the total body weight of healthy men and women, respectively [32]. WAT adipocytes are derived from myogenic factor 5-negative stem cells [33]. The first step of adipogenesis, MCE (Figure 1), links to the following characteristics: (i) active cell cycle; (ii) rearrangement of the cytoskeleton; and (iii) irreversible commitment to adipogenesis.

After adipogenesis induction, adipocyte precursors undergo several rounds of cell division before the cell cycle completely arrests. The commitment takes place in the G1 phase of cell cycle. This phase is characterized by a dramatic increase in PPARγ expression, which slows the cell cycle and determines the balance between proliferation and terminal differentiation [34], also studied in 3T3-L1 cells [35]. In the early phase of HM-induced differentiation, LiSa-2 cells enter the cell cycle for two rounds. In this early period, PPARγ and C/EBPα, master regulators of adipogenesis, activate the expression of genes connected with lipid metabolism and terminate MCE [35]. At the same time, the first hallmark of adipogenesis in LiSa-2 cells becomes visible, the accumulation of the cyclin-dependent kinase (CDK) inhibitor p27. CAND1 is required to eliminate SKP2 from CRL1^SKP2^, responsible for the ubiquitylation and subsequent degradation of p27 by the 26S proteasome (Figure 1). Associated with an increase in CAND1 and a decrease in SKP2, p27 accumulates during the adipogenic program of LiSa-2 preadipocytes [1]. In mouse 3T3-L1 cells, p27 is also controlled by CRL1^SKP2^, and the increase in p27 protein is likewise dependent on SKP2 suppression [36]. CRL4A^CDT2^ is another CRL involved in the degradation of p27 [37]. The particle was identified in HeLa and LiSa-2 cells as latent CSN^CSN7B^-CRL4A^CDT2^. During apoptosis in HeLa cells, the complex binds and stabilizes p27 [1]. In addition, it is necessary for cell cycle arrest in MCE during adipogenesis [1]. CSN^CSN7B^-CRL4A^CDT2^ loses its SR during the first hours of MCE [1] and contributes to the accumulation of p27 in the cytosol and in the nucleus at the end of MCE.

A cytoskeleton rearrangement occurs at the beginning of adipogenesis. Members of the RHO family of small GTPases regulate the actin dynamics in cells. They control microtubules, cell shape, movement, and differentiation [38]. A CRL3-dependent degradation of RHOA in mouse embryonic fibroblasts and in LiSa-2 cells using BTB domain-containing adapter/receptor for CUL3-mediated RHOA degradation protein 3 (BACURD3) as SR was shown (Figure 2) [6]. The ubiquitylation and degradation of RHOA by CSN^CSN7A^-CRL3^BACURD3^, connected with the rearrangement of the cytoskeleton, begin very early in adipogenesis and continue until TAD [6].

In the undifferentiated status, LiSa-2 preadipocytes accumulate large amounts of factors such as CHOP, an inhibitor of adipogenesis, which is quickly degraded after induction of the adipogenic program. CHOP forms a dominant negative heterodimer with C/EBPβ, preventing the transactivation ability of C/EBPβ, which results in the inhibition of adipogenesis [39]. It was shown that CHOP is ubiquitylated by the CRL3^KEAP1^ (KEAP1-KELCH-like ECH-associated protein 1) complex [1], most likely CSN^CSN7A^-CRL3^KEAP1^ (Figure 2). KEAP1 integration into the CRL3 complex is initiated by CAND1, available in large quantities in MCE [26]. Subsequently, CHOP is degraded by the 26S proteasome and disappears during TAD (Figure 1), one example of adipogenesis-specific proteolysis.

In summary, CSN-CRL complexes play a crucial role in the first phase of adipogenesis. CRL1 complexes, mostly involved in the cell cycle [40], are partially inactivated at the end of MCE. The accumulation of p27 is a hallmark of early differentiation in LiSa-2 cells, leading to cell cycle arrest. The cytoskeleton is rearranged, e.g., by CSN^CSN7A^-CRL3^BACURD3^ (Figure 2), for later transport of proteins and lipids during adipogenesis. Moreover, inhibitors of adipogenic differentiation like CHOP are degraded via CSN^CSN7A^-CRL3 using CAND1 and specific SRs (Figure 2). The process is similar in other model systems [41].

## 4. CSN^CSN7A^-CRL3^BTB^ Is Recruited to Lipid Droplets by RAB18 During TAD

**Figure 3:**
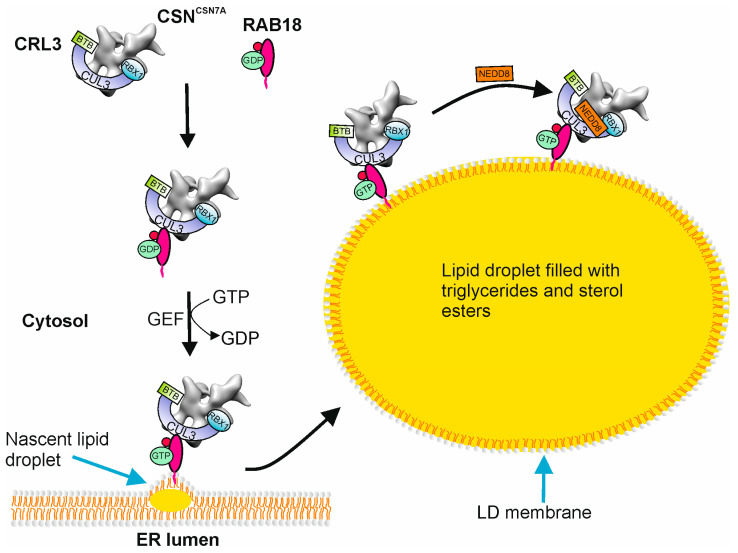
Recruitment of CSN^CSN7A^-CRL3^BTB^ to lipid droplet membranes by RAB18. Latent CSN^CSN7A^-CRL3^BTB^ is captured by GDP-RAB18 in the cytosol. The guanin nucleotide exchange factor (GEF) catalyzes the dissociation of GDP and binding of GTP to RAB18. GTP-RAB18 together with CSN^CSN7A^-CRL3^BTB^ is associated with the nascent lipid droplet (LD) on the endoplasmic reticulum (ER) membrane. On the LD membrane, the CSN^CSN7A^-CRL3^BTB^ is neddylated/activated. Whether the SR is linked to the CSN-CRL complex before recruitment or on the LD membrane is not yet clear.

Lipid droplets (LDs) are dynamic organelles derived from the endoplasmic reticulum (ER). LDs store neutral lipids, triglycerides, and cholesterol esters (Figure 3) [42] and are consumed when lipids are required.

Their hydrophobic core is bound by a monolayer phospholipid membrane originating from the ER [43]. Biogenesis of LDs in the ER membrane starts with lens formation, which is induced by sterol ester synthesis by acyl-CoA: cholesterol O-acyltransferases (ACAT1 and ACAT2) and triacylglycerols produced by diacylglycerol acyltransferases (DGAT1 and DGAT2). The next step is LD budding induced by the ER phospholipid composition. Proteins like seipin and PEX30 (YLR324W) are necessary for correct budding [44]. LDs grow through LD-LD fusion, through the transfer of triacylglycerol to LDs via the ER membrane, or through triacylglycerol synthesis on the LD (Figure 3). The LD proteome differs between cell types and comprises more than one hundred proteins in mammalian cells. Many proteins are involved in lipid synthesis and degradation. The perilipin (PLIN) family (PLINs 1–5) mostly maintains the structural integrity of LDs [9]. In general, proteins decorating LDs are subdivided into two classes: proteins derived from the ER and proteins originating from cytosol. ER proteins include the lipid biosynthesis enzymes glycerol-3 phosphate acyltransferase 4 (GPAT4) and diacylglycerol O-acyltransferase 2 (DGAT2), the acyl-CoA synthetase (ACSL3), the ER-associated degradation (ERAD) factors ancient ubiquitous protein 1 (AUP1) and ubiquitin-X domain adaptor 8 (UBXD8), the putative methyltransferase (AAM-B), caveolin-1 and caveolin-2, the hepatitis C virus (HCV) core protein (HSD17B11), and associated with LD protein 1 (ALD1) [9]. Proteins translated in the cytosol and targeted to LDs are, for example, PLINs. Many proteins such as PLIN1 [45], PLIN2 [46], adipose triglyceride lipase (ATGL) [47], and others are substrates of the UPS. Until now, it is mostly unclear how cytosolic proteins are transported to LDs.

RAB small GTPases affect the functions of LDs connected with the transfer of proteins. Recently, it was shown that the CSN^CSN7A^-CRL3 complex is recruited by RAB18 to LD membranes [1]. In LiSa-2 cells, RAB18 is phosphorylated upon stimulation of adipogenesis. Phosphorylation of RAB proteins has been described [48,49,50], but their functions remain unclear. We speculate that RAB18 is phosphorylated to elevate its affinity to CRL3. Phosphorylation of RAB18 begins upon induction of adipogenesis from day 1 and continues more than 2 weeks [1]. It strengthens the bond of RAB18 with the CSN^CSN7A^-CRL3 complex and thus enables recruitment to the LDs. The guanosine diphosphate (GDP)-bound inactive state (RAB18-GDP) localized in the cytosol binds the latent CSN^CSN7A^-CRL3 complex. The membrane-associated guanosine triphosphate (GTP)-bound active conformation (RAB18-GTP) fixes its cargo to the LD membrane (Figure 3). RAB18-GTP targets the entire latent CSN^CSN7A^-CRL3 to the LD membrane [1]. Interestingly, CRL3 is targeted to the surface of RAB7-positive endosomes. Unfortunately, in this case, the existence of CSN was not examined [51].

Whether appropriate BTB proteins are already attached or are on their way to or on the LD membranes assembled is not yet clear. It has been shown that KEAP1 is integrated into CRL3 in a CAND1-dependent manner for CHOP degradation [1] and that BACURD3 is an SR for the degradation of RHOA [6]. However, it is currently unclear when and where the SRs are installed and with which SRs the CSN^CSN7A^-CRL3 is equipped on LDs. Interestingly, in 3T3-L1 cells, KBTBD11 has been described as a BTB protein that is required for adipogenesis [52].

There is another exciting observation. The proteome of CULs includes DUBs like USP15 which specifically interacts with CSN7A [1]. In targeting CSN^CSN7A^-CRL3 to LD, RAB18 also transfers USP15 to the LD membrane as CSN^CSN7A-USP15^-CRL3^BTB^. Interestingly, USP15 has been identified on LDs. It interacts with PLIN2 [53], a protein responsible for the stability of the organelles. Under conditions of lipid deprivation, PLIN2 is poly-ubiquitylated and degraded by the 26S proteasome followed by reduced LD size and numbers [54]. We speculate that the function and stability of PLIN2 on LDs are controlled by the CSN^CSN7A-USP15^-CRL3^BTB^ complex.

Before CRLs come into action, they must be activated by neddylation. According to recent data in LiSa-2 cells, CUL3, as a component of CRL3, is neddylated at the LD membrane during adipogenesis (Figure 3) [6]. By inhibition of CRL3 neddylation using MLN4924 [6], LDs are not formed and adipogenesis is blocked. In other words, CSN^CSN7A^-CRL^BTB^ complexes loaded with the correct BTB protein and activated by neddylation are essential for LD formation and for TAD.

## 5. Selected Malfunctions of CSN^CSN7A^-CRL3^BTB^ and CSN^CSN7B^-CRL4A^DCAF^ Connected with Obesity

**Figure 4:**
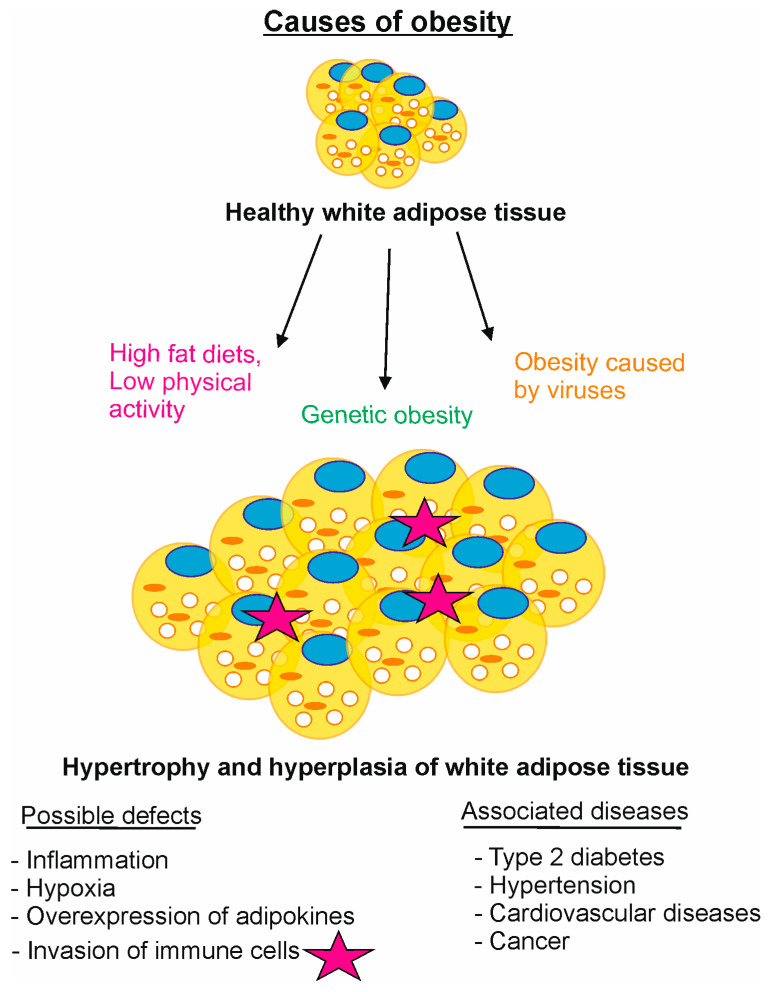
Selected causes of obesity. Malfunctions of CSN-CRL complexes can lead to obesity. High-fat diets with low physical activity, CSN-CRL gene defects, and infection subject healthy white adipose tissue to hypertrophy and/or hyperplasia. Possible side effects are inflammation, hypoxia, overexpression of adipokines, invasion of immune cells, and others. Associated are diseases like type 2 diabetes, hypertension, cardiovascular diseases, and cancer.

Deregulation of adipocyte differentiation has been associated with obesity [55], cardiovascular diseases [56], and cancer [57], risk factors for premature death. Defects of CSN-CRL complexes, main regulators of adipogenesis, can lead to obesity (Figure 4).

The current understanding of obesity is an imbalance of consumed and expended calories. At a body mass index (BMI) of 30 or greater, the patient is considered obese. Physical activity reduces the risk of obesity. Unfortunately, most of the population has an energy balance disorder. Under this condition, an accumulation of more fat leads to more/larger LDs, hypertrophy, and hyperplasia of white adipocyte cells (Figure 4). Currently, despite modest weight loss after lifestyle modifications and available medications for obesity, e.g., orlistat, phentermine/topiramate, lorcaserin, bupropion, and liraglutide [32], the disease cannot be cured. Progress has been made using glucagon-like peptide 1 (GPL-1) receptor agonists for the treatment of obesity, which, however, is embossed by severe side effects [58]. A hopeful drug is the bioactive natural product curcumin. It relieves obesity by blocking CSN^CSN7A^-CRL3 neddylation and inducing apoptosis [1]. In addition, curcumin or curcumin-derived compounds reduce serum lipid levels and modulate inflammation [59]. In severely obese individuals with a body mass index (BMI) of ~ 40 and above, bariatric surgery is the only evidence-based approach producing marked and sustainable weight loss. In studies with laparoscopic gastric bypass and sleeve gastrectomy, the average BMI decreased by more than 25% two years after surgery. In correlation with the BMI, VEGF decreased proportionally, which reduces the risk of cancer [60].

Recently, chromosomal defects causing obesity have been identified [61]. These defects include the deletion of chromosome region 17p11.2 (Smith Magenis syndrome; SMS). SMS is a typical example, which is a multiple congenital anomaly/intellectual disability syndrome associated with an interstitial deletion of chromosome band 17p11.2 [62]. In SMS, the COPS3/*CSN3* gene becomes haploinsufficient. The remaining CSN3 molecules form an intact CSN complex [62]. However, the reduced concentration of CSN might be responsible for defects of differentiation in the brain or fat tissue during embryogenesis and childhood. Recent observations show that besides neurodevelopmental disorders, >90% of patients with SMS are overweight or obese after 10 years of age [63]. Although many authors believe that retinoic acid-induced 1 (RAI1), localized in chromosome band 17p11.2, alone is responsible for the obese phenotype [64], we speculate that the reduced CSN could be an additional reason for the appearance of obesity after 10 years.

Interestingly, COPS7A/*CSN7A*, localized on 12p13.31, is synergistically expressed with all the other CSN subunit genes in regular tissues. The expression of COPS7B/*CSN7B*, mapped on 2q37.1, is regulated in a different way [65]. Children with an increase in the 12q13.31 region exhibit developmental delays, seizures, macrocephaly, eczema, and obesity [66]. Deletions of the 2q37 locus lead to brachydactyly mental retardation syndrome (BDMR). Patients exhibit overweight or obesity as well as cancer [67].

A monogenetic cause has been described for the heterozygous mutation of CSN2^WT/K70E^ in gene-manipulated mice [68]. CSN2 K70 is important for inositol hexakisphosphate (IP6)-mediated CSN-CRL binding, the glue between CSN and CRL [69]. Heterozygous mice with partially disrupted CSN-CRL display congenital hyperinsulinism and insulin resistance with risks of obesity. Homozygous CSN2^K70E/K70E^ knock-in mice as well as CSN2-null mice are embryonically lethal [68]. Interestingly, in the Human Obesity Gene Map, CSN genes are localized in chromosome regions with evidence for the presence of linkage with obesity-related phenotypes [68].

Research in past decades disclosed specialization of CRL families. Whilst CRL3 complexes fulfill pivotal functions in mammalian cell differentiation, CRL4 particles are often associated with chromatin and DNA repair [40]. *CUL3* was identified as a candidate gene for neurodevelopmental disorders like autism spectrum disorder [70]. Analyses suggest that a defect in *CUL3* affects multiple organs involved in the vascular, muscular, skeletal, and neurological systems [71]. *CUL3* is mapped to chromosome 2q36.2. A genetic cause of obesity has been described for *CUL3*. In a deletion of exon 9, amino acids 403–459 of CUL3 are missing (CUL3^Δ403–459^), causing pseudohypoaldosteronism type II, a familial form of hyperkalemia and hypertension [72]. The mutated CUL3^Δ403–459^ is unable to ubiquitylate WNK (With-No Lysine (K)) kinases or regulate Na^2+^/Cl^−^ transport connected with blood pressure adjustment. CUL3^Δ403–459^ loses its binding to CSN, causing self-ubiquitylation, and does not degrade substrates necessary for adipogenesis such as RHOA [72]. Unsurprisingly, pseudohypoaldosteronism type II, or so-called Gordon syndrome, is associated with obesity [73]. Patients possessing 13q34 microdeletions, the localization of the CUL4A gene, display clinical features including intellectual disability, mild facial dysmorphism, and obesity [74]. Recent data support the notion that high expression of CUL4A is associated with several types of cancer, including breast, lung, and bone cancer [75,76]. Interestingly, the anti-obesity factor WDTC1 (DCAF9), an SR of CRL4, reduces lipid production and suppresses adipogenesis via the CRL4^WDTC1^ ligase in 3T3-L1 cells [77]. Furthermore, in mice, the interaction between CSN-CRL can be modified by IP6, as mentioned above. IP6-dependent CSN-COP1 competition controls CRL4 activity, regulating glucose induced insulin secretion. Deregulation of CRL4-COP1 formation leads to obesity [68]. Findings support that supplementation and repair of deleted *CSN* subunit genes or *CUL3* as well as *CUL4* by CRISPR technology emerge as novel therapeutic approaches for the treatment of specific types of obesity.

Infection has recently received greater attention as an inducing factor of obesity (Figure 4) [78]. Six viruses are known to lead to obesity in animals [79]. Lately, human adenovirus 36 infection was related to obesity. In 3T3-L1 cells, adenovirus 36 stimulates the expression of genes involved in cell differentiation, including *CSN* and *CRL* genes, and increases lipid accumulation. The presence of adenovirus 36 in adipose tissue of overweight and obese humans has been confirmed [80,81]. The biological plausibility of obesity by viral infection rises, and the direct potential of some viruses to reprogram host metabolism toward increased lipid production and faster adipogenesis is presented. As soon as the infection is certain, work should be carried out on a vaccination against the corresponding virus.

## 6. Concluding Remarks

Discovery of the significant contribution of CSN-CRL complexes to adipogenesis represents another milestone in understanding the adipogenic program. The next step is to determine the missing SRs and to identify other CSN-CRLs involved in the process. Revealing wanted SRs will lead to the exact ubiquitylation reactions. Further, additional interacting key factors will be identified. Discovering crucial factors presents excellent targets for possible treatment. Since RAB18 has been identified as a CSN-CRL transporter to the LDs, the exact function of other RABs will be elucidated. It is assumed that RABs might also be outstanding treatment targets.

Obesity is an excellent example of how deeper knowledge of the involved process leads to better treatment options. While the inhibition of adipogenesis for the treatment of obesity is problematic so far, today, researchers are considering how inhibition of the process can alleviate obesity. Accurate knowledge of the CSN-CRL reaction offers outstanding opportunities for new therapies.

Future research will reveal how genetic treatment for specific forms of obesity might be appropriate. Along with the chemical influence of CSN-CRL complexes, genetic modifications might be suitable approaches. New insights will bring further answers for efficient treatment of obesity.

## Data Availability

No new data were created or analyzed in this study.
